# *Moringa oleifera* treatment increases Tbet expression in CD4^+^ T cells and remediates immune defects of malnutrition in *Plasmodium chabaudi*-infected mice

**DOI:** 10.1186/s12936-020-3129-8

**Published:** 2020-02-07

**Authors:** Jennifer Pilotos, Kadra Abdu Ibrahim, Chishimba Nathan Mowa, Michael Makokha Opata

**Affiliations:** Department of Biology, College of Arts and Sciences, Appalachian State University, 572 Rivers Street, ASU Box 32027, Boone, NC 28604 USA

**Keywords:** Malaria, Immunity, Effector T cells, Moringa, Malnutrition

## Abstract

**Background:**

Malaria is a worldwide problem that affects millions of people yearly. In rural areas where anti-malarial drugs are not easily accessible, many people use herbal treatments, such as *Moringa oleifera,* to treat a variety of diseases and ailments including malaria. While Moringa is reported to possess potent and curative anti-malarial properties, previous studies have mostly been restricted to assessment of parasitaemia. In this study, the effect of Moringa on malaria immunity in a murine model was investigated.

**Methods:**

Using a high dose (60 mg/mouse) for a short time (7 days) or low dose Moringa (30 mg/mouse) for a longer time (3 weeks), cytokine production, and Tbet expression by effector CD4^+^ T cells (Teff) were determined. Mice were also treated with Moringa after infection (curatively) or before infection (prophylactically) to determine the effect of the plant extract on parasitaemia and immunity. Given that Moringa also possess many nutritional benefits, the contribution of Moringa on malnourished malaria infected mice was determined. Malnutrition was induced by limiting access to food to only 4 h a day for 4 weeks, while control mice had unlimited access to mouse laboratory chow. All data was collected by flow cytometry and analysed using one-Way ANOVA or two tailed Student’s t test.

**Results:**

Moringa-treated mice had increased numbers of effector CD4^+^ T cells accompanied by an increase in Tbet expression compared to control untreated mice. Mice that were treated with Moringa curatively also exhibited increased effector CD4^+^ T cell numbers, IFN-gamma and TNF secretion. Interestingly, the mice that were treated prophylactically had significantly higher Tbet expression. In the absence of adaptive immunity, high parasitaemia was observed in the RAG1 knockout mice. The food limited mice (malnourished) had reduced numbers of CD4^+^ T cells, TNF proportions, and significantly greater Tbet expression compared to the control group. Supplementation with Moringa in the limited group slightly restored CD4^+^ T cell activation, IL-2, and IL-10 production.

**Conclusions:**

Taken together, these data suggest that Moringa treatment leads to increased CD4^+^ T cell activation, Th1 differentiation and production of pro-inflammatory cytokines after malaria infection. Thus, Moringa may be immunologically useful in the treatment of malaria and malnutrition. Further investigations are required to identify the active components in Moringa.

## Background

Malaria impacts millions of people yearly with a total of 217 million cases and 435,000 deaths reported in 2017 [1]. The sub-Saharan Africa region accounted for 90% of malaria cases and 92% of malaria deaths [2]. *Plasmodium falciparum*, the most deadly species of malaria parasites, accounted for almost all of the malaria-related mortality in sub-Saharan Africa [3]. Due to the high rates of mortality and morbidity caused by *P. falciparum* infection, use of anti-malarial drugs is essential to alleviate the disease [4]. In the recent past, there has been emergency of resistance towards many of the anti-malarial drugs, including chloroquine, sulfadoxine-pyrimethamine, quinine, piperaquine and mefloquine [5]; but traditional herbal treatments, such as *Moringa oleifera* have continuously been used to treat malaria as well as to alleviate malnutrition [6].

While progress has been made in the fight against malaria with recent approval of RTS,S/AS01 as a malaria vaccine; it only has 35.9% efficacy for the first year post-vaccination which decreases by 2.5% in the fourth year and 4.4% in the seventh year post-vaccination [7]. With this low efficacy and increased resistance in anti-malarial drugs [5], combination therapies of anti-malarial drugs with artemisinin are used [8]. Although these combination therapies have allowed for treatment of the resistant strains, recent epidemiological studies have shown the emergence of artemisinin-resistant *P. falciparum* in Thailand, Laos, and Cambodia [9, 10]. This growing resistance calls for the development of effective anti-malarial treatments as wells as combination therapies and *Moringa oleifera* could be a promising candidate.

Moringa, also known as drumstick tree, is an edible plant of the *Moringaceae* family which is cultivated in the sub-Himalayan tracts of Pakistan, India, Bangladesh, Afghanistan [11] and in many parts of Africa [12]. All parts of the plant (seeds, leaves, bark, roots, sap, and flowers) can be consumed and possess a variety of attributes that are used in traditional medicine [13].

Moringa can be consumed raw, cooked, or dried into a powder [14], then used as a nutritional supplement or to treat a variety of ailments including scurvy, purgation, headaches, fevers, otitis, sore throat, bronchitis, and eye infections [15]. Other studies have shown that Moringa possess a variety of properties including anti-diabetic, anti-inflammatory, anti-cancer [16], anti-ischemic [17] and even anti-plasmodial properties [15]. Of interest to the present study, Moringa has been shown to possess immune boosting properties [18], and potent curative/suppressive effects on parasite burden in *Plasmodium* infection [8, 19, 20].

Studies performed to quantify the biochemical properties and efficacies of Moringa have yielded positive results. For instance, a study performed by Dondee *et al.* showed a dose dependent suppressive effect on parasite growth of up to 90% and an 80% reduction in parasite burden [19]. Olasehinde et al. [20] also observed this suppressive effect in their study that employed both crude ethanolic and an n-hexane Moringa leaf extracts. They found that the crude ethanolic extract inhibited parasitaemia by 74.7 to 95.6% and *n*-hexane extract inhibited by 59.3 to 87.9%. Moringa has also been found to be effective in combination therapies with artesunate according to Somask et al. [8]. When combined with artesunate, Moringa suppressed parasitaemia up to 91% in a dose dependent fashion compared to 50% seen with artesunate alone [8]. Studies to determine the immune response by Sijabat et al. [18] showed that a combination therapy of methanolic Moringa extract and artemisinin not only reduced parasite burden, but also increased the percentage of CD4^+^ T cells in a dose dependent manner.

Moringa is also consumed to treat malnutrition in many malaria endemic areas [11, 21]. It has been shown to be a high source of fiber, protein, calcium, iron, vitamin C, and carotenoids [22]. Nutrition studies have shown that Moringa provides more than 7 times the amount of Vitamin C in oranges, 10 times the amount of Vitamin A in milk, 9 times the amount of protein in yogurt, 15 times the amount of potassium found in bananas, and 25 times the amount of iron in spinach [23]. Human studies have shown that 8 grams of Moringa leaf powder can provide a toddler with 14% of the protein, 23% of the iron, and 40% of the calcium recommended daily [14]. Also, studies investigating Moringa’s effect on malnutrition have shown that Moringa supplementation improved protein energy malnutrition in 70% of grade II malnourished children, and 60% of grade I malnourished children [24].

Malnutrition has been implicated in approximately half of the deaths reported in children under the age of five [25] and is common in many rural malaria endemic areas due to limited access to a variety of nutritious foods [26, 27]. The effect of malnutrition on malaria infection has been the topic of much controversy over the years. Some studies suggest that malnutrition exacerbates the already life-threating symptoms of malaria infection, leading to greater malaria morbidity and mortality [28], while others suggest that malnutrition reduces parasitaemia, leading to less disease severity [29, 30]. Studies in cohorts of undernourished and stunted children have shown greater susceptibility to malaria infection [31], because of a notable reduction in T lymphocytes, impaired antibody production, decreased complement production, and atrophy of the thymus and other lymphoid tissues [32]. These observations have been supported by studies comparing stunted and wasted children. Both stunted and wasted children exhibited reduced anti-*P. falciparum* IgG antibodies compared to controls [30, 33]. In another study, the investigators found that controlled trials of vitamin A and zinc supplementation led to significant reduction in clinical malaria attacks in previously malnourished children [28]. These studies suggest that malnutrition has a direct link with malaria immunity and/or severity.

Although the parasite inhibiting properties and nutritional benefits of Moringa have been studied, Moringa’s effect on malaria immunity and malnutrition is still poorly understood. Therefore, the current study aimed to investigate the effect of Moringa treatment on the immune response in mice infected with *Plasmodium chabaudi* strain AS, a mouse species that induces chronic infection similar to *P. falciparum*. The beneficial effects of Moringa in the treatment of malaria in malnourished mice was also determined.

## Methods

### Mice and parasite

C57BL/6 mice used in this study were obtained from Harlan laboratories and breeding colonies were maintained at the Appalachian State University animal facility under a 12:12 light/dark cycle. All mice were cared for under the guidelines set by the IACUC (protocol 17-04).

The rodent malaria parasite *P. chabaudi*, an established mouse model for human malaria, was used in this study to mimic the chronic nature of the human parasite strain *P. falciparum*. The parasite was a kind gift from Dr. Robin Stephens at the University of Texas Medical Branch, with permission from Dr. Jean Langhorne (Francis and Crick Institute, UK).

### Preparation of Moringa pellets

Moringa leaves were a kind gift from Dr. Chishimba Nathan Mowa in the Department of Biology at Appalachian State University, or obtained from Natural Market (Boone, NC). Otto’s cassava flour was obtained from EarthFare grocery store (Boone, NC). Cassava flour was used in this study to make Moringa pellets as it contains empty calories and held the leaf powder together. Mice were treated with low, high or nutritional doses depending on the experimental question as outlined in the results section.

#### Low dose Moringa pellets

Moringa pellets were made using 10.10 g of cassava flour and 370 mg of Moringa leaf powder (30 mg of Moringa per mouse for a 3-day supply). These were mixed with sterile DI water to form pellets and ~ 2 g peanut butter was mixed in for flavour enhancement. The control pellets were made using 10.37 g of cassava flour and ~ 2 g of peanut butter.

#### High dose Moringa pellets

Moringa pellets were made using 10.10 g of cassava flour and 910 mg of Moringa leaf powder (60 mg of Moringa per mouse for a 3-day supply). These were mixed with water to form pellets and ~ 2 g peanut butter was added for flavour enhancement. The control pellets were made using 11.00 g of cassava flour and ~ 2 g of peanut butter.

#### Nutritional dose Moringa pellets for nutritional supplementation

Moringa pellets were made every 3 days using 20.10 g of cassava flour and 500 mg of Moringa leaf powder per mouse under a sterile hood. These were mixed with sterile distilled water to form pellets and ~ 4 g of peanut butter mixed in for flavour enhancement. Twelve pellets were formed and allowed to air dry. Mice were given four pellets daily along with standard mouse chow for 3 days.

### Treatment of mice

#### Moringa experiments

Adult 8-week old female C57Bl/6 mice were utilized in these experiments to determine the effect of Moringa on the generation of malaria immunity. Mice were fed Moringa pellets daily for 7 or 23 days before infection (pre-infection) or after infection (post-infection). Control mice were fed pellets made of cassava and peanut butter without Moringa for 7 days. In prophylactic/curative studies, mice were fed for 3 weeks before and throughout infection (prophylactic) or 9 days after infection (curative). Mice were infected with a 1 × 10^5^ dosage of *P. chabaudi* AS and sacrificed at day 9 post-infection (p.i.) to harvest spleen cells for effector time points. To determine the effect of Moringa on parasitaemia, a mix of adult male and female immunocompromised Recombinant Activating Gene knockout (RAG1 KO) mice were fed Moringa or control pellets for 3 weeks. The mice were infected with 1 × 10^5^ iRBCs. Weights and parasitaemia were determined until day 12 p.i..

#### Food limitation experiments

Adult 8-week old C57Bl/6 mice were utilized in these experiments to determine the effect of food limitation induced malnutrition on malaria immunity. Food limitation was performed by only allowing the malnourished (Mal) mice to access standard laboratory mouse chow for 4 h daily, while control mice were given unlimited access to food (24/7). Mice were weighed weekly before infection or every other day after infection with a 1 × 10^5^ dosage of *P. chabaudi* AS. Experimental mice were sacrificed at day 9 p.i. to determine immune response in spleen cells.

#### Food limitation and Moringa experiments

Adult 8-week old C57BL/6 mice were utilized in these experiments to determine if Moringa could remediate the effects of food limitation induced malnutrition on malaria immunity. Food limitation was performed by only allowing the malnourished group access to standard mouse chow for 4 h daily while control mice were given unlimited access to food. A third group of the malnourished mice were supplemented with Moringa pellets as a nutritional supplement after food was removed for 20 h. All mice were weighed weekly before infection or every other day after infection with a 1 × 10^5^ dosage of *P. chabaudi* AS and sacrificed at day 9 p.i. to determine immune response in spleen cells. Control groups given Moringa, but not infected were included to determine the effect of Moringa on naïve cells.

### Flow cytometry analysis

Spleens were collected in ISCOVEs media and mashed through mesh screens to obtain single cell suspensions. Cells were incubated with red blood cell (RBC) lysis buffer to lyse red blood cells which was stopped by adding media. The cells were then resuspended in complete ISCOVEs media. The cells were counted using a hemocytometer and an aliquot was taken for staining with extracellular molecules using fluorochromes CD11a-FITC–clone M17/4, (Biolegend, San Diego, CA), CD44-PE–clone PE, CD4-PE-Cy5–clone GK1.5, and CD62L-PE-Cy7–clone MEL-14 (Tonbo Biosciences, San Diego, CA), to determine T cell activation. For intracellular cytokine staining, aliquots for each sample from the counted cells were stimulated in vitro with a cell stimulation cocktail (Tonbo Biosciences) for 5 h. After 5 h of stimulation, cells were stained with CD4-FITC–clone GK1.5 and incubated for 40 min in the fridge before fixation with 2% Paraformaldehyde. Cells were then permeabilized using perm/wash buffer (Tonbo Biosciences, San Diego, CA), and incubated with Fc block for 20 min followed by a 40-min incubation with IFNγ PE–clone XMG1.2, and TNF-PE-Cy7–MP6-XT22, or IL-2-PE–JES6-5H4, and IL-10 FITC–clone JES5-16E3 (all from Biolegend). Data was collected on an FC500 (Beckman Coulter, Indianapolis, IN) flow cytometer and analysed by FlowJo (Ashland, OR).

### Determining parasitaemia

Parasite burden was determined using thin blood smears obtained by bleeding the tail of the mice between days 3 to 9 for wildtype or days 3 to 12 for RAG1 KO mice post-infection with *P. chabaudi* AS. The slides were stained with Diff-Quik and parasites were counted by microscopy in 10 to 50 different fields depending on the parasite load and day of infection. To determine percent parasitaemia, the number of infected red blood cells was divided by the total number of red blood cells in all counted fields. The outcome was multiplied by 100 as shown in the formula below.

$$\% Parasitaemia = \frac{iRBC}{{\left( {Total RBC} \right)}} \times 100$$

### Data analysis

All flow cytometry data were analysed using the FlowJo software (Ashland, OR). The number of cells was determined by the counts taken using the haemocytometer. The average and standard error of the mean for all groups were determined using Microsoft excel and calculated cell numbers. Prism GraphPad 8 software (La Jolla, CA) was used to run One-way ANOVA or Student’s two-tailed t tests and generate the graphs.

## Results

### Short-term treatment with low dose Moringa pellets leads to decreased effector T cell numbers in post-infection treated mice

**Fig. 1:**
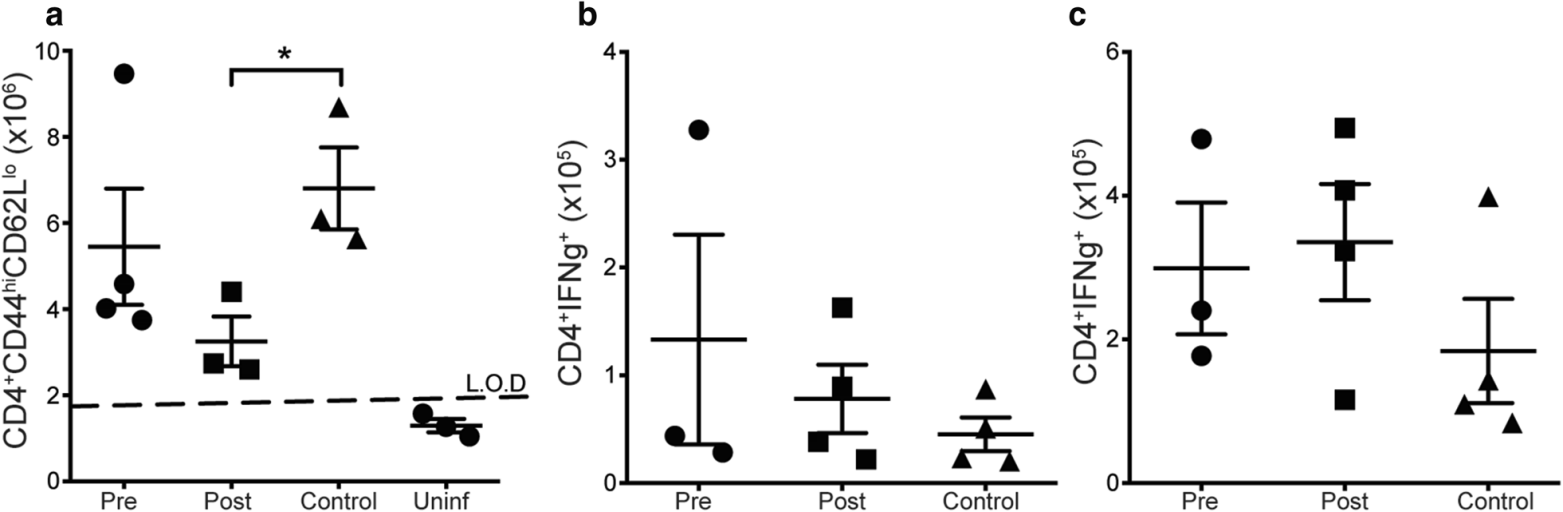
Administration of Moringa pellets after infection decrease the number of activated effector CD4^+^ T cells. C57BL/6 mice were fed low dose Moringa pellets (30 mg/mouse) for 7 days pre or post-infection with 1x10^5^ dose of *P. chabaudi AS* and control mice were given control pellets (no Moringa) prior to infection. Graphs show **a** effector T cells identified using CD4^+^CD44^hi^CD62L^lo^, **b** IFNγ and **c** TNF producing CD4^+^ T cell numbers determined using intracellular cytokine stimulation assay and analysed using flow cytometry. Data represent 4 mice per group from a representative experiment of 2 independent experiments. Error bars represent SEM and significance was determined by One-Way ANOVA followed by comparisons between individual groups. *p < 0.05. *Pre* Moringa treatment before infection, *Post* Moringa treatment after infection, *Control* No Moringa treatment but infected, *Uninf* Moringa treatment but not infected control, *L.O.D* limit of detection

Given that Moringa has been reported to possess potent suppressive and curative anti-malarial properties, the effect of low dose Moringa (30 mg/mouse) on immune response was investigated. Moringa pellets were fed to the mice for 7 days pre- or post-infection with *P. chabaudi*. All mice were then sacrificed at day 9 p.i. to determine the numbers of activated effector CD4^+^ T cells and cytokine secretion using the gating strategy shown in Additional file 1: Figs. S1 and S2. There was a significant reduction in the number of activated effector CD4^+^ T cells in mice treated post-infection with Moringa pellets (Fig. 1a). No significant differences were observed in the percentage or number of pro-inflammatory cytokines IFNγ and TNF between the groups, although there were trends towards increased numbers of IFNγ and TNF secreting CD4^+^ T cells in the Moringa treated mice compared to the control group (Fig. 1b, c). Taken together, these data suggest that low dose Moringa treatment may decrease T cell activation if administered after infection, but with no significant effects on cytokine secretion.

### Short term treatment with high dose Moringa increase Tbet expression in pre-infection treated mice

**Fig. 2:**
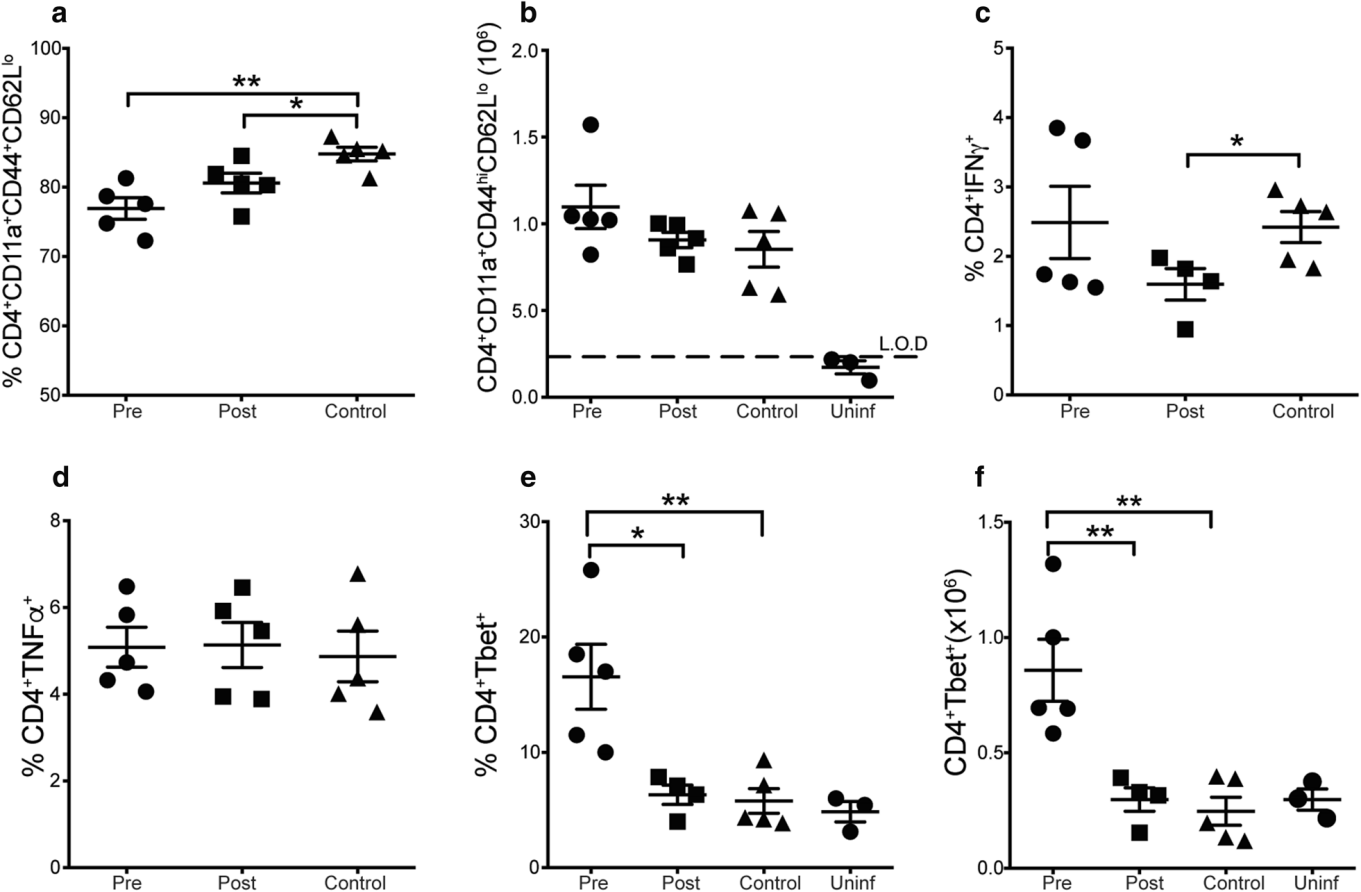
Tbet expression is increased in mice fed high dose Moringa before infection. Mice were fed high dose Moringa pellets (60 mg/mouse) for 7 days pre-infection or post infection, including respective controls without Moringa treatment. Mice were then infected with a 1 × 10^5^ dose of *P. chabaudi AS*, and sacrificed at day 9 post-infection. Graphs show **a** Percent and **b** number of activated effector CD4^+^ T cells identified as CD4^+^CD44^hi^CD62L^lo^. Percent of **c** IFNγ and **d** TNF producing CD4^+^ T cells determined using intracellular cytokine staining and analysed using flow cytometry. **e** Percent and **f** number of Tbet expression by CD4^+^ T cells determined using flow cytometry. Data represents an average of 5 mice per group from a representative experiment of 2 independent experiments and error bars represent SEM. Significance was determined by two-tailed t test. *p < 0.05, **p < 0.01. *Pre* Moringa treatment before infection, *Post* Moringa treatment after infection, *Control* No Moringa treatment but infected, *Uninf* Moringa treatment but not infected control, *L.O.D* limit of detection

People in developing nations consistently take Moringa in its raw form and, therefore, it is likely that they consume it at a higher dose than the recommended 100 g as a nutritive supplement for humans [34]. To test the effect of high dose Moringa consumption, mice were given high dose Moringa pellets-60 mg per mouse per day for 7 days pre- or post-infection with appropriate controls which were fed cassava control pellets. The mice were sacrificed at day 9 p.i. and percentage and numbers of effector CD4^+^ T cells were evaluated, as well as pro-inflammatory cytokines (IFNγ and TNF) and Tbet. There was a significant reduction in the percentage of activated effector CD4^+^ T cells in both treated groups compared to the control mice, but there was no significant difference in the cell numbers among all the groups (Fig. 2a, b). Interestingly, there was a significant decrease in the proportion of IFNγ production in the post-treated mice compared to the control mice (Fig. 2c), but no difference was observed in the percent of TNF among the groups (Fig. 2d).

Production of IFNγ and TNF is associated with Tbet expression, a master regulator of Th1 effector CD4^+^ T cell subset [35]. Therefore, the effect of high dose Moringa treatment on Tbet expression was determined using the gating strategy in Additional file 1: Fig. S3. There was an increase in both the percentage (Fig. 2e) and number (Fig. 2f) of CD4^+^ T cells expressing Tbet in the mice that were given Moringa before infection. Taken together, these results suggest that Moringa treatment before infection increases Tbet expression.

**Fig. 3:**
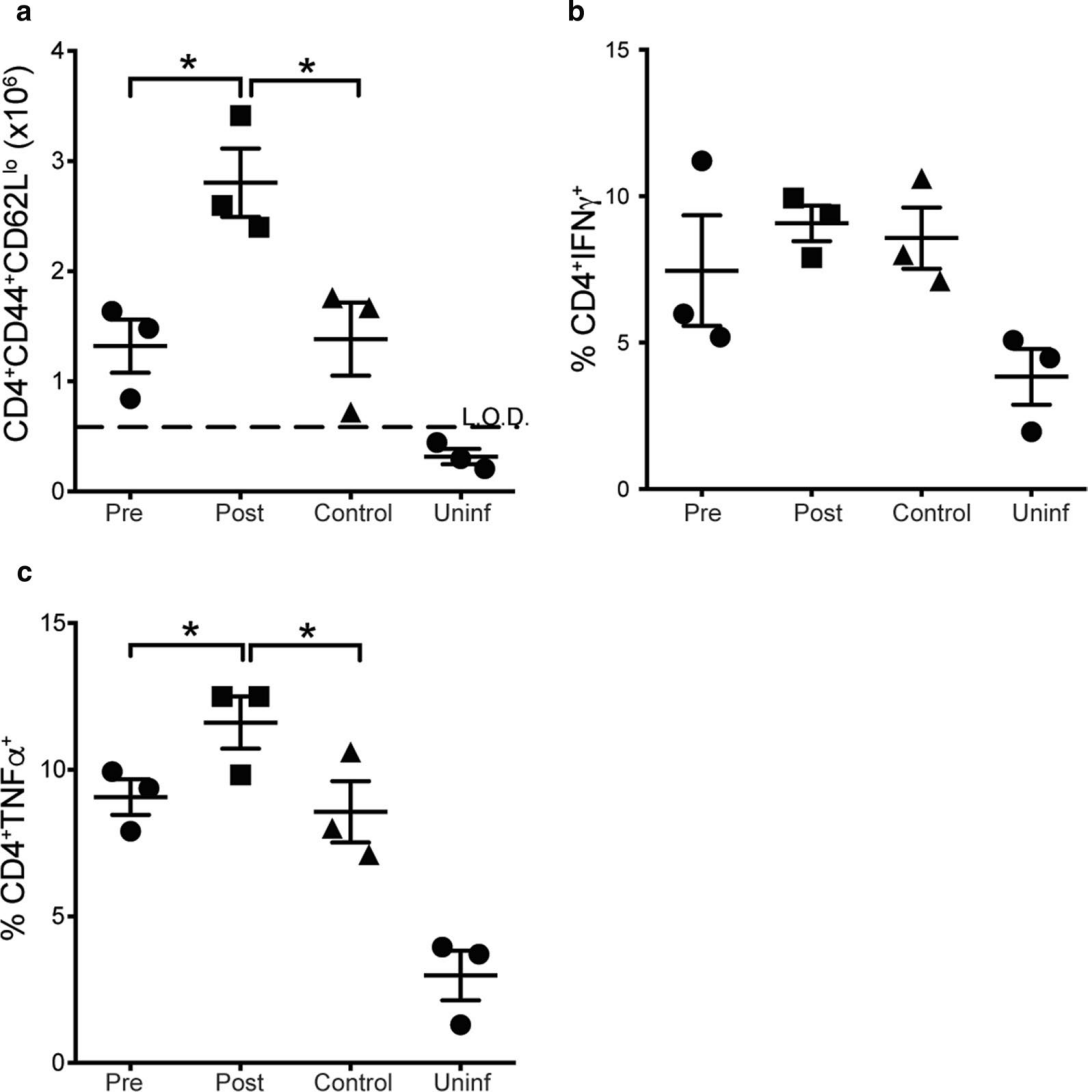
Long-term treatment with Moringa pellets increase the number of activated effector and TNF secretion in post infection treated mice. C57BL/6 mice were fed Moringa pellets for 3 weeks pre-infection or post-infection; control mice were fed control pellets. Mice were infected with a 1 × 10^5^*P. chabaudi AS*. Mice were sacrificed 6 weeks later at day 23 post infection. **a** Number of activated effector CD4^+^ T cells identified using CD4^+^CD44^hi^CD62L^lo^ and analysed by flow cytometry. Percentage of CD4^+^ T cells producing **b** IFNγ and **c** TNF, respectively. Data represent 3 mice per group from a representative experiment of 2 independent experiments. Error bars represent SEM and significance was determined by One-Way ANOVA followed by comparisons between individual groups. *p < 0.05. *Pre* Moringa treatment before infection, *Post* Moringa treatment after infection, *Control* No Moringa treatment but infected, *Uninf* Moringa treatment but not infected control, *L.O.D* limit of detection

### Long-term treatment with Moringa pellets leads to increased numbers of activated effector and TNF producing CD4^+^ T cells in post-infection mice

As most individuals in malaria endemic areas consistently consume Moringa for a long time and given the reduction in CD4^+^ T cell activation observed in Fig. 1a, the effect on the immune response of low dose treatment with Moringa for a long time was determined. To accomplish this, Moringa pellets were fed to the mice for 3 weeks pre- or post-infection with *P. chabaudi* with appropriate controls that were fed normal laboratory chow along with cassava control pellets. The mice were then sacrificed after a total of 6 weeks and the percentages and number of activated effector CD4^+^ T cells and pro-inflammatory cytokine secretion were determined. Interestingly, when Moringa was given for a long duration (3 weeks), there was an increase in the number of activated effector CD4^+^ T cells in mice treated post-infection compared to the pre-infection treated mice and the control groups (Fig. 3a), but no significant difference was observed in the percentages of these cells. TNF secretion was also increased, but no statistical difference was observed in IFNγ secretion (Fig. 3b, c) between the groups. Taken together with previous data, this may suggest that while short term Moringa treatment had a minimal effect on CD4^+^ T cell activation, long-term treatment significantly affects activation of CD4^+^ T cells when parasite load decrease.

### Prophylactic or curative consumption of Moringa leads to reduced parasitaemia and increased CD4^+^ T cell activation and pro-inflammatory cytokine secretion

**Fig. 4:**
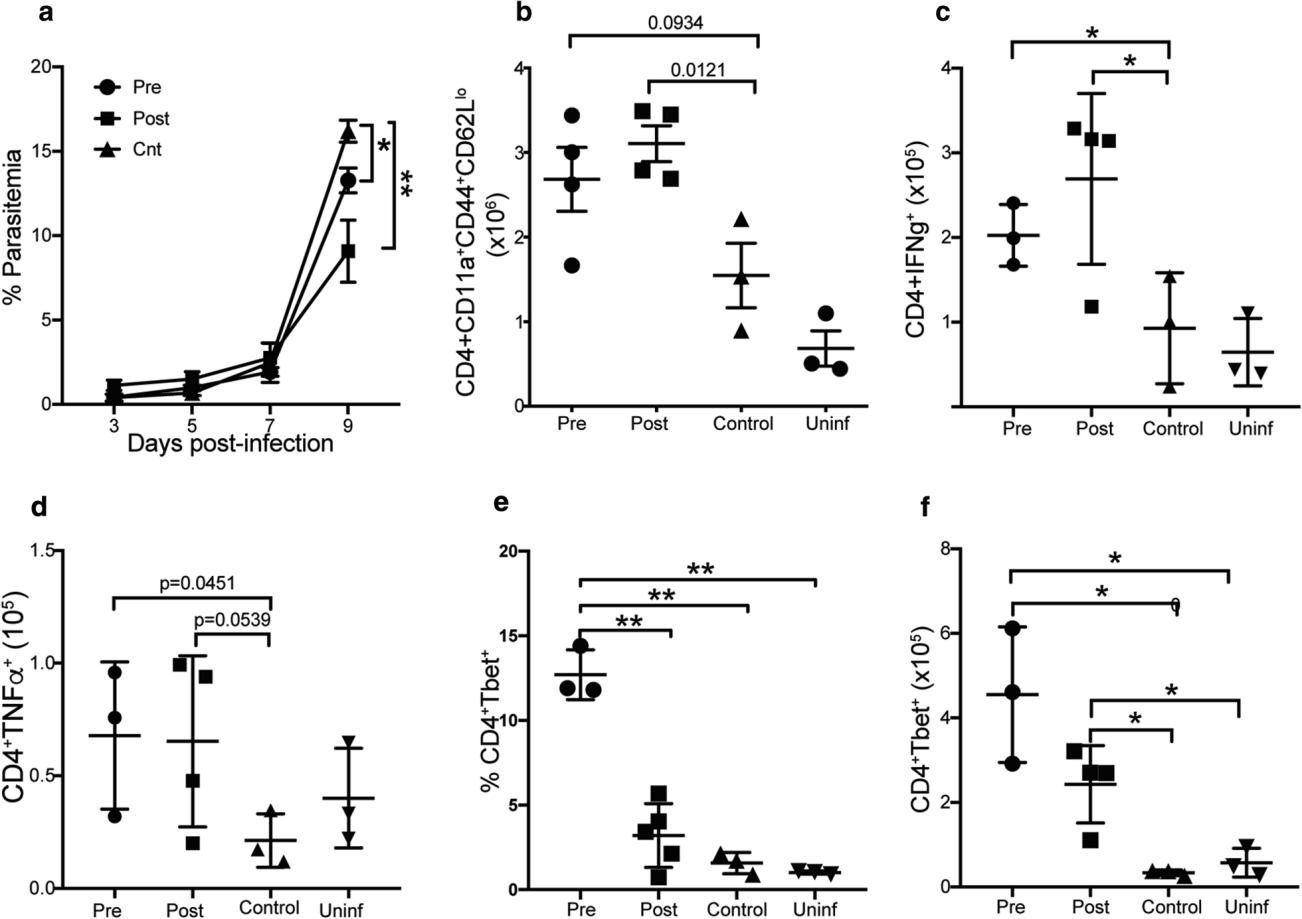
Prophylactic/curative Moringa treatment increased activated effector CD4^+^ T cell numbers, cytokine secretion, and Tbet expression in post-infection mice. Mice were fed high dose Moringa pellets (60 mg/mouse) for 7 days pre-infection or post-infection, including respective controls without Moringa treatment. Mice were infected with a 1x10^5^ dose of *P. chabaudi* AS, and sacrificed at day 9 post-infection. Graphs show **a** Percent parasitaemia, **b** Number of activated effector CD4^+^ T cells identified as CD4^+^CD11a^+^CD44^hi^CD62L^lo^. Numbers of **c** IFNγ and **d** TNF producing CD4^+^ T cells determined using intracellular cytokine staining and analysed using flow cytometry. **e** Percent and **f** number of Tbet expression by CD4 T cells determined using flow cytometry. Data represents an average of 5 mice per group from a representative experiment of 2 independent experiments and error bars represent SEM. Significance was determined by One-Way ANOVA followed by comparisons between individual groups. *p < 0.05, **p < 0.01. *Pre* Moringa treatment before infection, *Post* Moringa treatment after infection, *Control* No Moringa treatment but infected, *Uninf* Moringa treatment but not infected control, *L.O.D* limit of detection

**Fig. 5:**
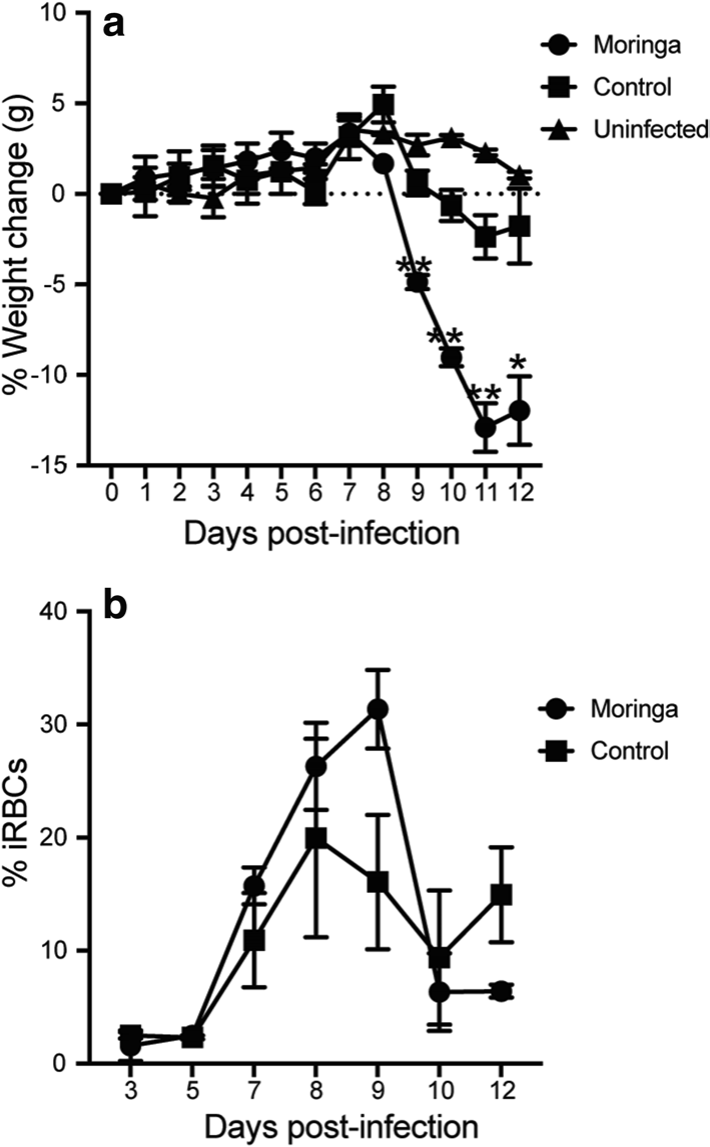
Moringa treatment increase parasitaemia in RAG1 KO mice. Adult RAG1 KO mice were fed Moringa or control pellets for 3 weeks, then infected with 1 × 10^5^*P. chabaudi*. Graphs show **a** Weights and **b** parasitaemia monitored over time. Data represent an average of 3–4 mice per group and error bars represent SEM including uninfected control mice. Significance was determined by two-tailed t test with 95% confidence, *p < 0.05, **p < 0.01

Traditionally, individuals who consume Moringa for its anti-plasmodial properties consume it either before being sick as a prophylactic or after getting sick for cure (curatively) [36]. To better mimic these conditions, mice were fed with low dose Moringa pellets (30 mg/mouse) prophylactically for 3 weeks prior to and throughout infection or curatively after infection with *P. chabaudi*. Blood smears were taken for 9 days to determine parasitaemia, and mice were sacrificed to assess CD4^+^ T cell immune response. Consistent with literature, a significant decrease in parasite load was observed at d9 p.i. (peak of infection) in the Moringa treated mice compared to the untreated controls (Fig. 4a). Upon sacrifice, the proportions and number of activated effector CD4^+^ T cells, cytokine production and Tbet expression were determined. There were significantly higher numbers of activated effector CD4^+^ T cells in curative (post) treated group compared to controls. The prophylactic treated mice had a trend towards increased cell numbers but did not reach statistical difference (Fig. 4b). There were also higher proportions and numbers of CD4^+^ T cells secreting pro-inflammatory cytokines IFNγ and TNF in Moringa treated mice compared to control mice (Fig. 4c, d). Strikingly, the prophylactic treated group had significantly higher proportions of Tbet expression (Fig. 4e). Both groups of Moringa treated mice exhibited increased numbers of CD4^+^ T cells expressing Tbet compared to control mice (Fig. 4f). In all cases, mice fed on Moringa but were not infected had T cells, cytokine or T bet expression below the limit of detection (L.O.D.), suggesting that Moringa has no detectable effect on naïve CD4^+^ T cells. To test if Moringa had a direct influence on the parasite, RAG1 KO mice were fed with Moringa or control pellets for 3 weeks. These mice were then infected with 1 × 10^5^ iRBCs followed by determination of weights and parasite load up to day 12 post-infection. The Moringa treated mice drastically lost weight starting on day 8 p.i (Fig. 5a), this correlated with high parasitaemia in the Moringa treated mice compared to the mice fed on the Control pellets (Fig. 5b). Taken together, these data suggest that continuous consumption of Moringa may boost the immune response and facilitate control of parasitaemia in healthy individuals with competent CD4^+^ T cells.

### Malnutrition induced Tbet expression, but reduced effector CD4^+^ T cell activation

**Fig. 6:**
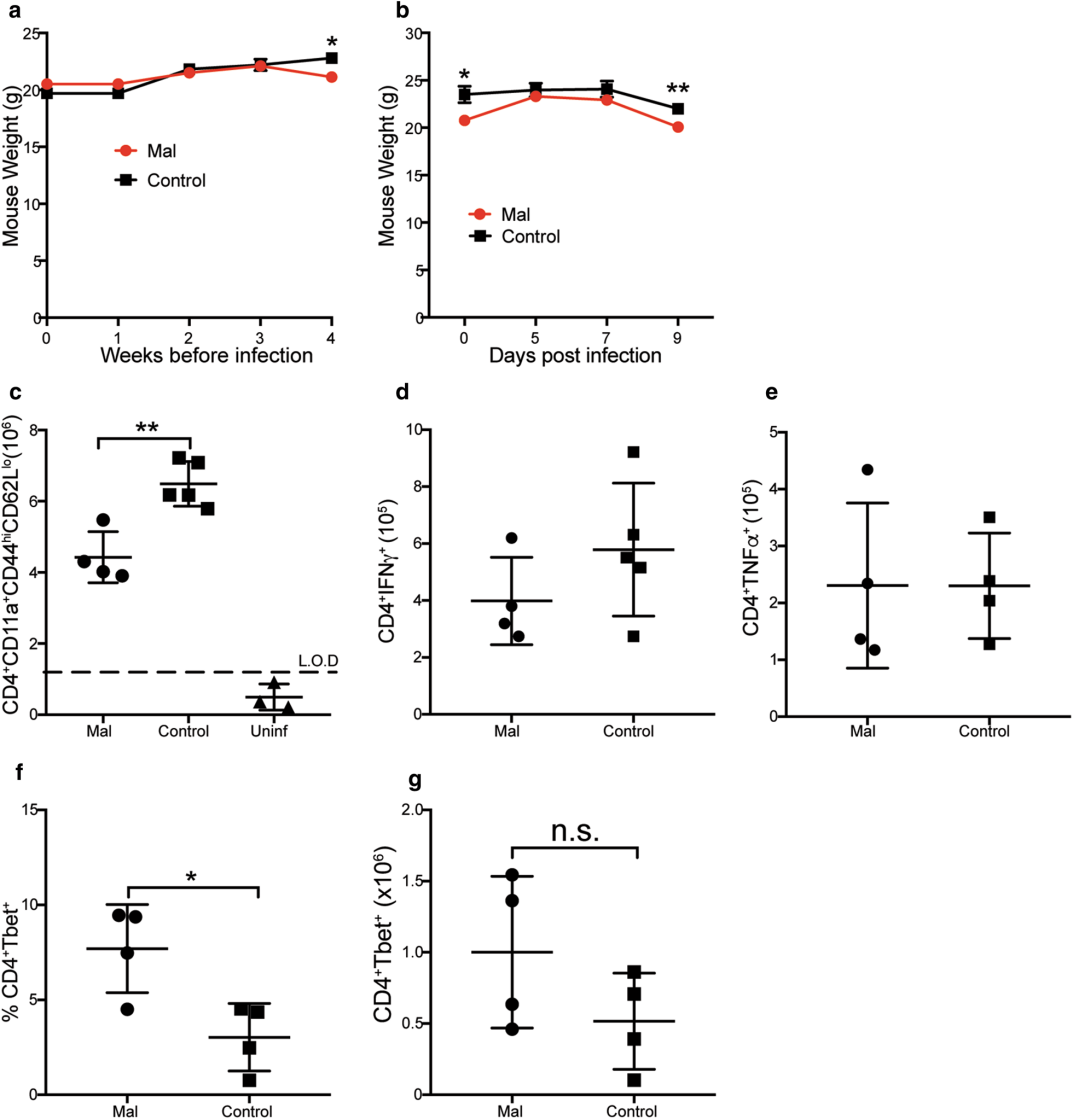
Malnourished mice express high Tbet, but reduced activated effector CD4^+^ T cells. Adults C57Bl/6 mice were moderately malnourished through food limitation by only allowing mice access to food for 4 h daily for 3 weeks, while controls had unlimited access to food. All mice were infected with a 1 × 10^5^ dose of *P. chabaudi AS* and sacrificed at day 9 post-infection. Summary graphs shows mouse weight in grams over **a** weeks post diets, **b** days post-infection. Numbers of **c** activated effector CD4^+^ T cells **d** IFNγ and **e** TNF producing CD4^+^ T cells. **e**, **f** Percent and number of Tbet expressing CD4^+^ T cells. Data represents an average of 5 mice per group and error bars represent SEM. Significance was determined by two-tailed t test with 95% confidence, *p < 0.05. *Mal* malnourished, *Uninf* uninfected control mice

**Fig. 7:**
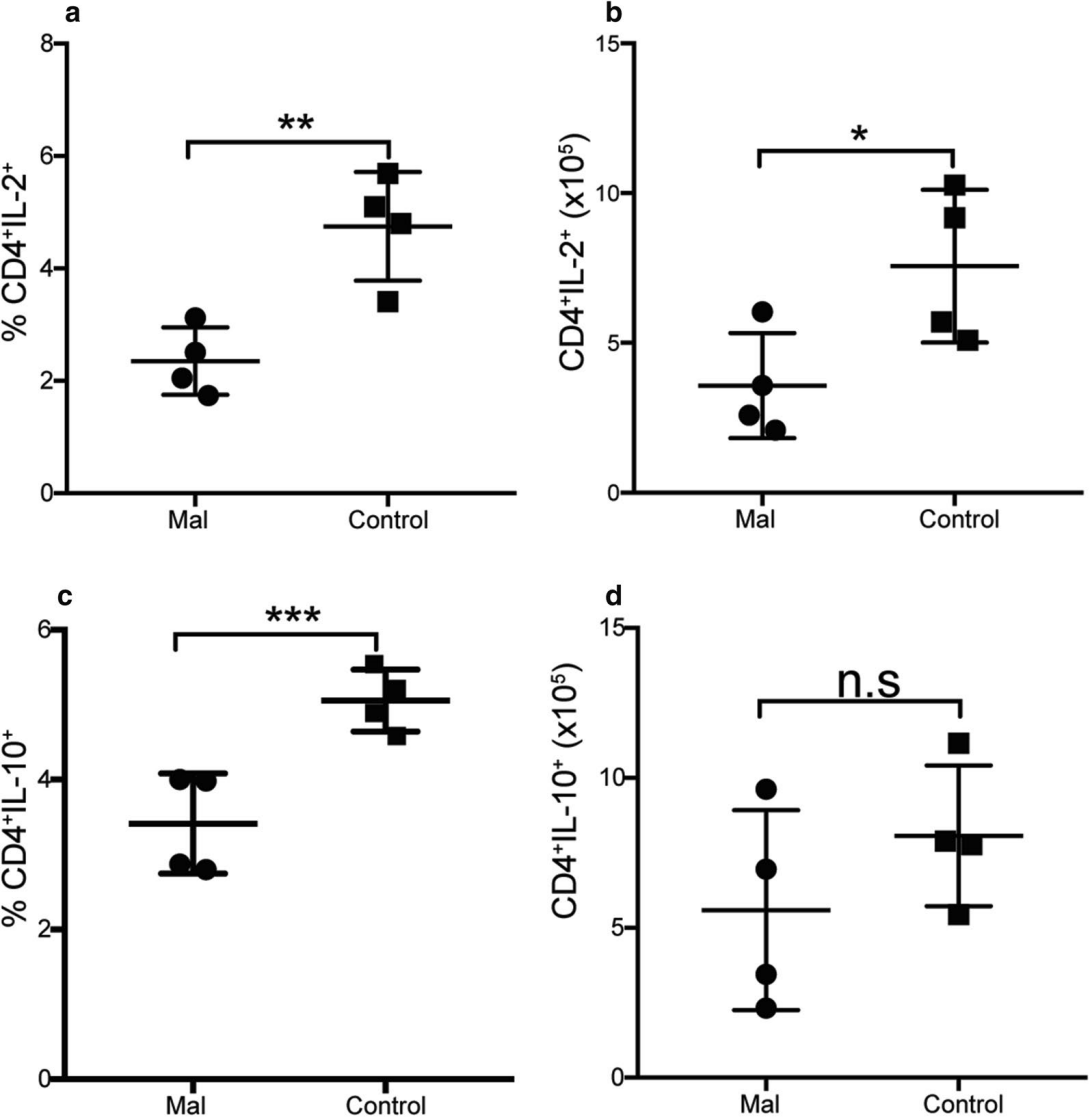
CD4^+^ T cells from malnourished mice produce less IL-2 and IL-10. Adults C57Bl/6 mice were malnourished through food limited by only allowing mice access to food for 4 h daily for 3 weeks, while controls had unlimited access to food. All mice were infected with a 1 × 10^5^ dose of *P. chabaudi AS* and sacrificed at day 9 post-infection. Summary graphs show **a** Percent and **b** Number of IL-2 producing CD4^+^ T cells. **c** Percent and **d** numbers of IL-10 producing CD4^+^ T cells determined using intracellular cytokine staining. Data represents an average of 5 mice per group and error bars represent SEM. Significance was determined by two-tailed t test with 95% confidence, *p < 0.05, **p < 0.001, **p < 0.0001, *n.s* no significance difference, *Mal* malnourished

Malnutrition is a common issue in many low income, malaria endemic areas and children are the most affected by malnutrition and malaria infection [25, 27, 28]. To understand how malnutrition affects CD4^+^ T cell immunity against malaria infection, a moderate malnutrition model was developed by food limitation. To induce the moderate malnutrition, a group of mice was limited to standard lab mouse chow (4 g per mouse) to 4 h daily, while control mice had unlimited access to food. The mice were exposed to this pattern of feeding for 4 weeks, and weighed weekly to monitor mouse health. The mice were then infected with a 1 × 10^5^ dose of *P. chabaudi,* and sacrificed at day 9 p.i. to determine CD4^+^ T cell numbers, Tbet expression, and cytokine secretion profiles. The mice that were kept on the malnourished pattern of feeding had no difference in weight until week four, at which time moderate malnutrition had been induced (Fig. 6a). After infection, there was no difference in weight until day 9 p.i., which is also the peak of infection (Fig. 6b). There were reduced numbers of effector CD4^+^ T cells observed in the malnourished group compared to control mice (Fig. 6c). There was a slight reduction in IFNγ that did not reach statistical difference, but there was no difference in TNF production between the malnourished and the control mice (Fig. 6d, e). Surprisingly, the malnourished mice exhibited increased expression of Tbet compared to controls (Fig. 6f, g). Due to a decrease in activated CD4^+^ T cells, the effect of malnutrition on IL-2 production, as IL-2 is important for T cell proliferation was determined. There was a significant decrease in both percent and number of IL-2 producing CD4^+^ T cells (Fig. 7a, b). Th1 cells in *Plasmodium* infection are characterized with IL-10 production to modulate pathology. Therefore, the effect of moderate malnutrition on IL-10 production was determined. Just like IL-2, there was a reduction in the percent of IL-10, but there was no difference in cell numbers (Fig. 7c, d). Taken together, these results indicate that malnutrition alters CD4^+^ T cell activation and IL-2 production, but has no effect on pro-inflammatory cytokines (IFNγ and TNF) or anti-inflammatory IL-10.

### Nutritional supplementation with Moringa increased CD4^+^ T cell activation in malnourished food limited mice

**Fig. 8:**
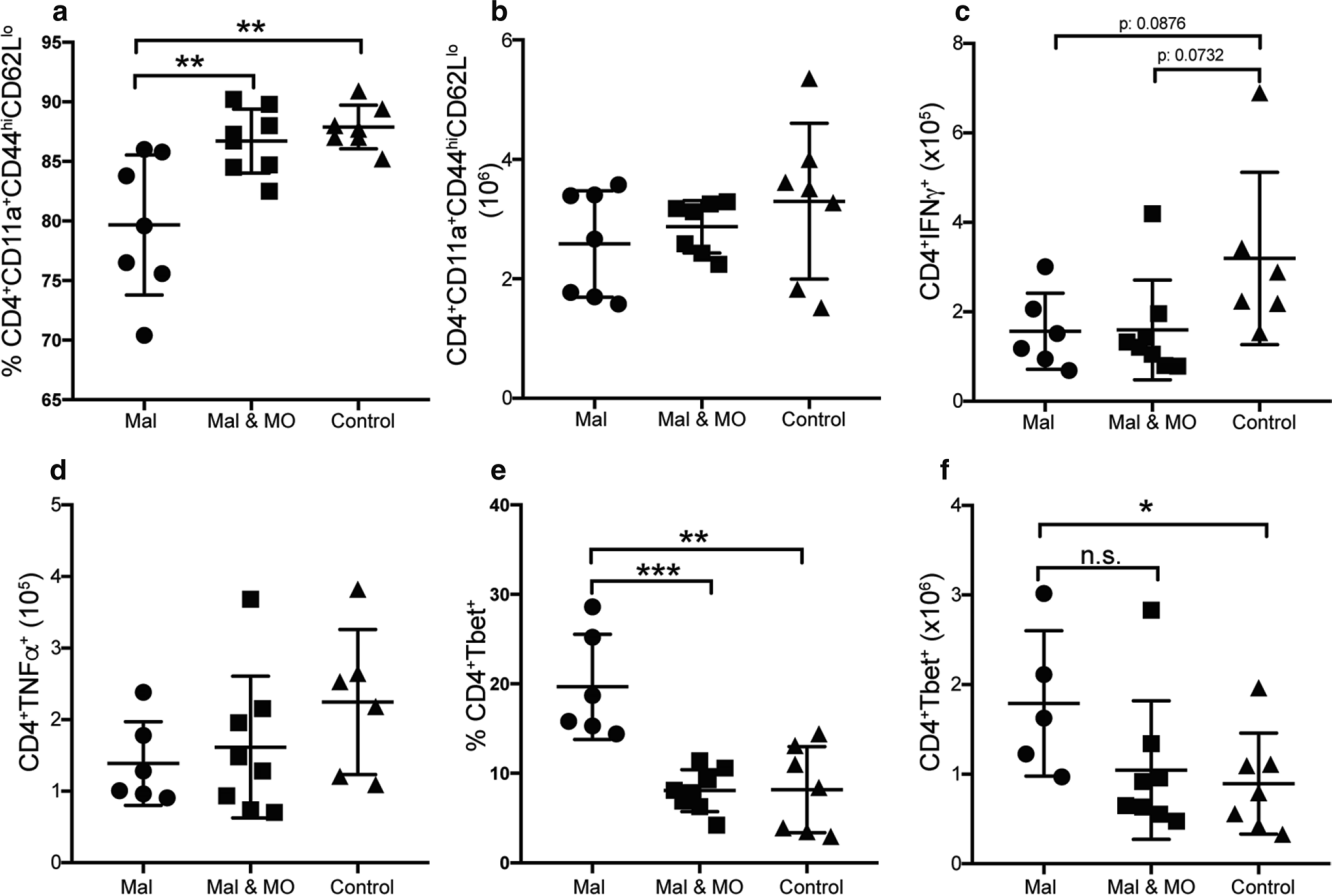
Nutritional supplementation with Moringa increased effector T cell proportions in malnourished mice. Adult C57Bl/6 mice were malnourished by food limitation by allowing mice access to food for 4 h daily for 4 weeks prior to infection. A second group of malnourished mice were supplemented with Moringa (500 mg per mouse), while controls had constant supply of food. All mice were infected with a 1 × 10^5^ dose of *P. chabaudi* AS and sacrificed at day 9 post-infection. Summary graphs show **a** Percent and **b** numbers of effector CD4^+^ T cells identified by CD4^+^CD44^hi^CD62L^lo^. Numbers of **c** IFNγ and **d** TNF producing CD4^+^ T cells. **e** Percent and **f** number of Tbet expression on CD4^+^ T cells determined using flow cytometry. Data represents an average of 5 mice per group and error bars represent SEM. Significance was determined by two-tailed t test with 95% confidence, *p < 0.05, **p < 0.01. *Mal* malnourished, *Mal & MO* malnourished supplemented with Moringa

**Fig. 9:**
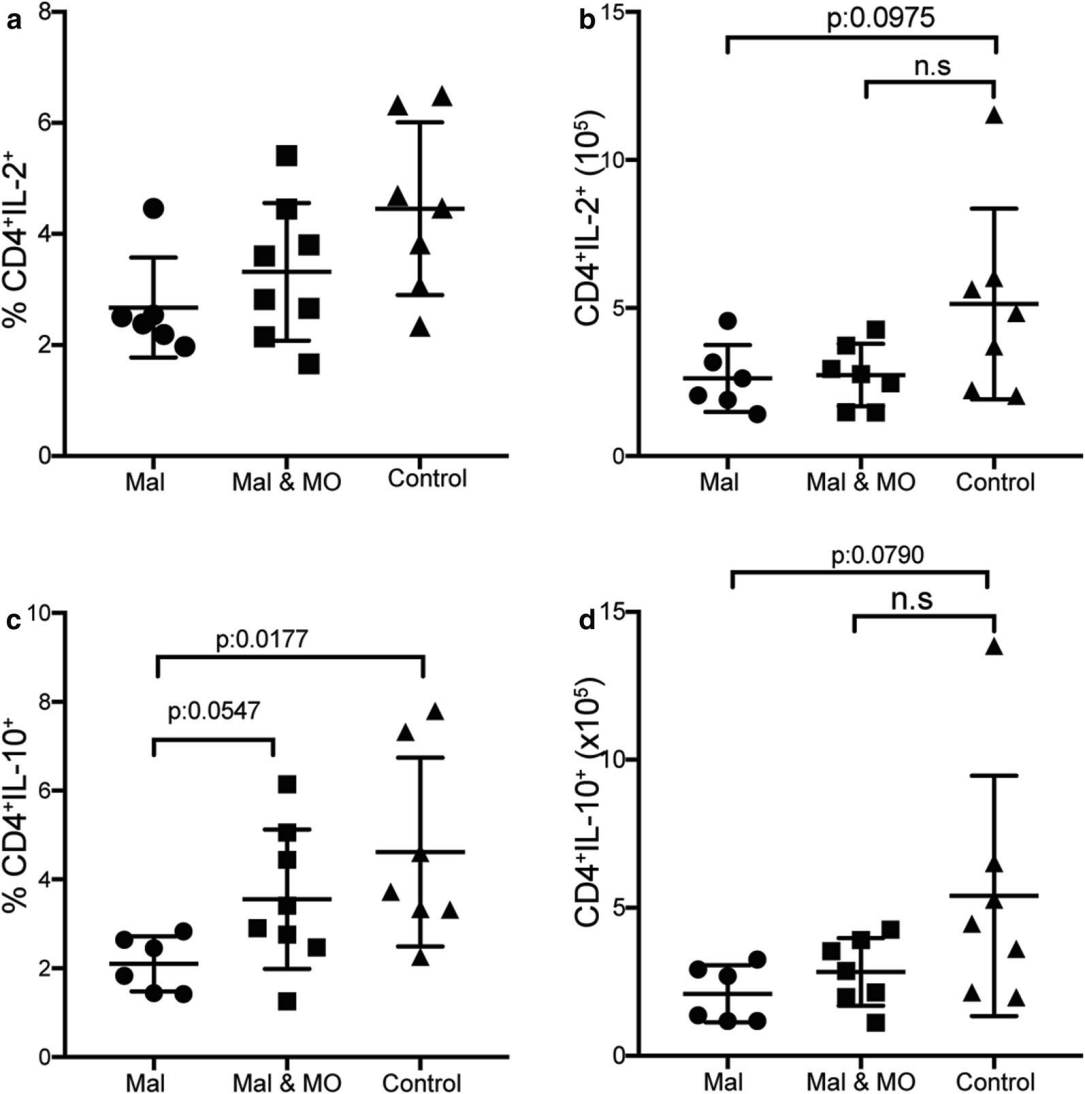
Nutritional supplementation with Moringa slightly increase percent of IL-2 and IL-10. Adult C57Bl/6 mice were malnourished by food limitation by allowing mice access to food for 4 h daily for 4 weeks prior to infection. A second group of malnourished mice were supplemented with Moringa (500 mg per mouse), while controls had constant supply of food. All mice were infected with a 1 × 10^5^ dose of *P. chabaudi AS* and sacrificed at day 9 post-infection. Summary graphs show **a** Percent and **b** Number of IL-2 producing CD4^+^ T cells. **c** Percent and **d** numbers of IL-10 producing CD4^+^ T cells determined using intracellular cytokine staining. Data represents an average of 5 mice per group and error bars represent SEM. Significance was determined by two-tailed t test with 95% confidence. *n.s* no significance difference *Mal* malnourished, *Mal & MO* malnourished supplemented with Moringa

The reduced numbers of activated effector CD4^+^ T cells in malnourished mice was expected, as studies suggest that malnutrition has detrimental effects on immune response [24, 28]. Given that Moringa is rich in protein [34] and the current study showed that it increases CD4^+^ T cell numbers and proportions, Moringa’s potential to remediate the immune suppressive effects of malnutrition was determined. Using the malnutrition model of 4 h food restriction, two sets of mice were malnourished. One group of the malnourished mice was given nutritional Moringa pellets upon food removal for the remaining 20 h. There was a slight increase in activated CD4^+^ T cell proportions in the Moringa supplemented mice compared to limited mice, with trends towards increased cell numbers that did not reach statistical significance (Fig. 8a, b). Supplementation had no effect on both IFNγ and TNF (Fig. 8c, d), interestingly, Moringa supplementation reduced Tbet expression in the malnourished mice to same levels as the control group (Fig. 8e, f). Unexpectedly, there was no increase in IL-2 secretion observed in the Moringa supplemented mice (Fig. 9a, b), but IL-10 secretion was slightly increased (Fig. 9c, d). Taken together these results suggest that Moringa supplementation may ameliorate some of the immune suppressive effects induced by malnutrition upon malaria infection such as CD4^+^ T cell activation.

## Discussion

Drug resistance to malaria infections has been observed for years and combination therapies have become the normal course of treatment to combat this fact. Quinine and artemisinin are used in combination therapies with other anti-malarial drugs to effectively treat malaria [5]. Despite their effectiveness, the investigation of other potential anti-malarial drugs or adjuncts is greatly needed, as there are new cases of resistance reported to the combination therapies [37]. Most of these medications are plant derivatives. Indigenous people in malaria endemic areas also use other plants and herbs to treat various illnesses including malaria. One such plant is Moringa, widely used in southern Nigeria for its medicinal and nutritional properties [38]. Many individuals consume Moringa as a raw vegetable frequently added to foods. The leaves can also be boiled in water, making an extract, that is used to treat malaria, stomach pains, high blood pressure, stroke, rheumatism, and to ease labour symptoms with a > 70% fidelity level [38].

While there are limited studies on the effect of Moringa on T cell activation, the current report shows that mice given a low dose of Moringa for 3 weeks after infection had increased numbers of activated CD4^+^ T cells in the spleens in response to the *Plasmodium* infection. Treatment with Moringa was also accompanied with increased proportions of TNF. Interestingly, immune suppressive tendencies were observed when Moringa was administered to the mice in some cases, and there were no big changes in CD4^+^ T cell activation. Similar inflammatory inhibition characteristics have been observed with LPS [39]. While this was a notable discrepancy in this study, a decrease or increase in cytokine production or CD4^+^ T cell activation was based on dose and duration of treatment. For example, when mice were treated with a high dose for a short time, there was no effect on activated effector CD4^+^ T cells in the treated mice, but long-term treatment increased CD4^+^ T cell activation and cytokine secretion capability. Notably, while there were no significant variabilities in cell activation or cytokine secretion in the mice treated before infection with high dose pellets, Tbet expression was increased in the effector cell numbers. Thus, it can be hypothesized that increased Tbet expression observed in the mice treated before infection may be responsible for the lack of a decrease in IFNγ observed in this group. Higher Tbet expression in this case promotes Th1 cytokine secretion, which could explain the similar proportions of cytokine secretion seen among the groups as reported by others [11].

Moringa has long been suggested to possess some anti-inflammatory properties [11] and murine studies using Moringa leaves have shown a significant reduction in experimentally induced inflammation [40]. The immune response to malaria is characterized by a robust Th1 inflammatory response [41]. This robust response is triggered by the expression of transcriptional regulator Tbet [42] which is upregulated when the T cells are primed to secrete IFNγ and other Th1 related cytokines. Unlike most parasitic infections, such as helminth, which rely on eosinophils, *P. falciparum* is capable of suppressing eosinophilia [43], and is characterized by secretion of Th1 cytokines IFNγ and TNF [44]. Recent studies have shown production of IL-10 by Th1 cells to regulate the inflammatory response [41]. In some cases, a surge in inflammation due to malaria infection leads to severe or moderate malaria, specifically due to higher levels of circulatory pro‐inflammatory cytokines, such as TNF and IL‐6, but IL-10 regulates the outcome [45]. As Moringa is reported to have anti-inflammatory effects [46], it could reduce this surge, hence protect from severe malaria.

When mice were treated curatively or prophylactically to mimic field scenarios where people take Moringa continuously, parasitaemia was inhibited in both cases of Moringa treatment, whether administered for cure or for prophylaxis. This was accompanied by significant activation of CD4^+^ T cells, cytokine secretion and Tbet expression, a master regulator for Th1 CD4^+^ T cell subset. The consistently higher expression of Tbet in the pre-infection treated group could indicate that early treatment with Moringa programs the cells to be more Th1 biased. Despite programming a Th1 biased environment for the CD4^+^ T cell in Moringa pre-treated group, high levels of macromolecules in crude Moringa [47] may inhibit pro-inflammatory cytokine secretion which would explain why the pre-treated mice have increased Tbet expression but not inflammatory cytokine secretion. The increase in activated CD4^+^ T cells can enhance macrophage activation that phagocytosis of parasites, hence reduced parasitaemia in the Moringa fed mice.

A biochemical safety study on the micro and macronutrients present in Moringa was performed by Asiedu-Gyekye and colleagues using an *in vivo* murine model [47]. In their studies to access the macromolecules, they treated mice with a subacute single dose of 5000 mg/kg and a range of 0 mg/kg to 1000 mg/kg (40, 80, 200, and 1000 mg/kg) for 14 days. White blood cell counts increased by 52.5% compared to controls in their single high dose as well as in their 40 mg/kg and 80 mg/kg dosages [47]. In comparison, this study was performed at 30 mg/animal (low dose) and 60 mg/animal (high dose) and only treated for 7 days or 9 days, compared to 14 days by the other researchers. Based on these observations, it can be assumed that immune suppression may occur early in Moringa treatment at suboptimal levels (as our results show), but when treated for longer periods of time at a dosage of 40 mg/kg to approximately 80 mg/kg, there is immune stimulation as shown by Asiedu-Gyekye et al. [47]. But due to use of unpurified Moringa leave extracts, the levels of macromolecules cannot be determined in the current study, this could be a contributing factor to some of the discrepancies observed in the various treatment groups in our data.

In the nutritional studies, food limitation induced malnutrition decrease CD4^+^ T cell activation, but there was no notable difference in pro-inflammatory cytokine production. Accompanying the reduction in effector CD4^+^ T cell numbers, was a significant reduction in IL-2 and IL-10 cytokines in the malnourished mice. IL-2 is important for T cell proliferation while L-10 reduces inflammation. With reduced effector T cell numbers and low IL-2, it is possible that malnutrition affect proliferation as well, but this was not tested in this current study. Although this reduction in T cell activation was not surprising as many human studies have shown notable reduction in immune response to malaria in malnourished cohorts [25, 27, 33], a reduction in pro-inflammatory cytokines was expected as reported in human studies where plasmatic levels of signature Th1 cytokines IL-2 and IFNγ were lower in malnourished children [48]. Lack of a reduction in inflammatory cytokines could be due to increased Tbet expression that was observed in the CD4^+^ T cells from the malnourished mice.

The many micro and macronutrients that Moringa is known to possess prevent malnutrition [49, 50]. Similarly, many studies have reported medicinal use of Moringa as reviewed by Gopalakrishnan et al. [34]. In the current study, Moringa supplementation in the malnourished mice was shown to increase the proportions of activated CD4^+^ T cells and numbers and the general immune response of Moringa supplemented mice bares many similarities to the control mice. Even though Moringa seemed to increase CD4 mediated immune response, specifically enhancing Tbet expression, in some instances there was a suppression of inflammatory cytokines. The discrepancies in these studies may be associated with many reports suggesting that some of the nutrients in Moringa are detrimental to the body or promote parasite growth. For example, one study reported that treatment with the aqueous extract of Moringa in rats affect the liver leading to increased alanine aminotransferase, aspartate aminotransferase, alkaline phosphatase and acid phosphatase, which may cause liver damage [51]. Similarly, Moringa contain vitamin C (ascorbic acid) which increase parasite growth as it is taken up into the red blood cells [52, 53]. While the liver damaging enzymes were not tested for in this study, high parasitaemia was observed when Moringa was given to immunocompromised mice that do not have B and T cells. Suggesting that indeed CD4^+^ T cells and the adaptive immunity is important for the beneficial effects of *Moringa oleifera*.

## Conclusions

While Moringa is extensively believed to have anti-plasmodial properties by inhibiting parasite growth, these data suggest that it may enhance CD4^+^ T cell activation as well. Increased T cell numbers are important for helper function and parasite clearance by the host’s immune system. There was an observable decrease in parasitaemia upon Moringa treatment; this decrease was accompanied by increased cytokine secretion and Tbet expression in mice that were treated with Moringa before (prophylactic) or after (curative) infection. Immune stimulating properties were also observed in infected malnourished mice that were supplemented with Moringa. When immunocompromised mice that lack T cells were treated with Moringa, then infected with malaria, there was a significant growth in the parasites compared to control mice, confirming that Moringa can promote parasite growth [52], but in the presence of CD4^+^ T cells, the parasites may be controlled. Therefore, these results suggest that the use of Moringa prophylactically or curatively in healthy people is beneficial in the control of malaria disease and treatment of malnutrition.

## Supplementary information

**Additional file 1: Figure S1.** Flow cytometer gating strategy for Effector CD4^+^ T cells (Teff) activation. Spleen cells were obtained from *P. chabaudi* infected (top panels) or uninfected Moringatreated mice (bottom panels) and stained for CD4, CD11a, CD44, CD62L. Lymphocytes were identified by side and forward scatter (left panel). Recently activated CD4 T cells were identified as CD4^+^CD11a^+^ (middle panel) and effector T cells were identified as CD44^hi^CD62L^lo^ (right panel). **Figure S2.** Flow cytometer gating strategy for IFNγ secreting CD4 T cells. Spleen cells were obtained from *P. chabaudi* infected (top panels) or Moringa treated uninfected (bottom panels) mice. Cells were stimulated for intracellular stained as explained in the “Materials and methods” section. Lymphocytes were identified by side and forward scatter (left panels) and IFNγ and TNFa secreting CD4 T cells were identified by CD4^+^IFNγ^+^ or CD4^+^TNFa^+^ (two panels to the right). **Figure S3.** Flow cytometer gating strategy for Tbet expression by CD4^+^ T cells. Spleen cells were obtained from adult *P. chabaudi* infected (top panels) or uninfected Moringa-treated mice (bottom panels). Cells were surface stained for CD4, fixed in 2% paraformaldehyde then permeabilized using permeabilization/FOXP3 buffer and intracellularly stained for Tbet. Lymphocytes were identified by side and forward scatter (left panels), followed by Tbet expression by CD4^+^ T cells identified as CD4^+^Tbet^+^ (middle and right panels).

## Data Availability

The datasets used and/or analysed during the current study are available from the corresponding author on reasonable request.

## Abbreviations

p.i: post-infection
L.O.D: limit of detection
SEM: standard error of the mean
Mal: malnourished
Teff: effector T cells
IFNγ: interferon gamma
TNF: tumour necrosis factor
IL: interleukin
Tbet: T-box transcription factor
TGFβ: transforming growth factor beta
Treg: regulatory T cells
ANOVA: analysis of variance
IACUC: Institutional Animal Care and Use Committee
