# Supplementary material for: Moringa oleifera treatment increases Tbet expression in CD4+ T cells and remediates immune defects of malnutrition in Plasmodium chabaudi-infected mice
Source: Malar J. 2020 Feb 7;19:62. doi: 10.1186/s12936-020-3129-8 (PMC7006207; doi:10.1186/s12936-020-3129-8)
Supplement: Supplementary file 1 — Additional file 1: Figure S1. Flow cytometer gating strategy for Effector CD4+ T cells (Teff) activation. Spleen cells were obtained from P. chabaudi infected (top panels) or uninfected Moringatreated mice (bottom panels) and stained for CD4, CD11a, CD44, CD62L. Lymphocytes were identified by side and forward scatter (left panel). Recently activated CD4 T cells were identified as CD4+CD11a+ (middle panel) and effector T cells were identified as CD44hiCD62Llo (right panel). Figure S2. Flow cytometer gating strategy for IFNγ secreting CD4 T cells. Spleen cells were obtained from P. chabaudi infected (top panels) or Moringa treated uninfected (bottom panels) mice. Cells were stimulated for intracellular stained as explained in the “Materials and methods” section. Lymphocytes were identified by side and forward scatter (left panels) and IFNγ and TNFa secreting CD4 T cells were identified by CD4+IFNγ+ or CD4+TNFa+ (two panels to the right). Figure S3. Flow cytometer gating strategy for Tbet expression by CD4+ T cells. Spleen cells were obtained from adult P. chabaudi infected (top panels) or uninfected Moringa-treated mice (bottom panels). Cells were surface stained for CD4, fixed in 2% paraformaldehyde then permeabilized using permeabilization/FOXP3 buffer and intracellularly stained for Tbet. Lymphocytes were identified by side and forward scatter (left panels), followed by Tbet expression by CD4+ T cells identified as CD4+Tbet+ (middle and right panels). [file 12936_2020_3129_MOESM1_ESM.pdf]

Additional File Figures

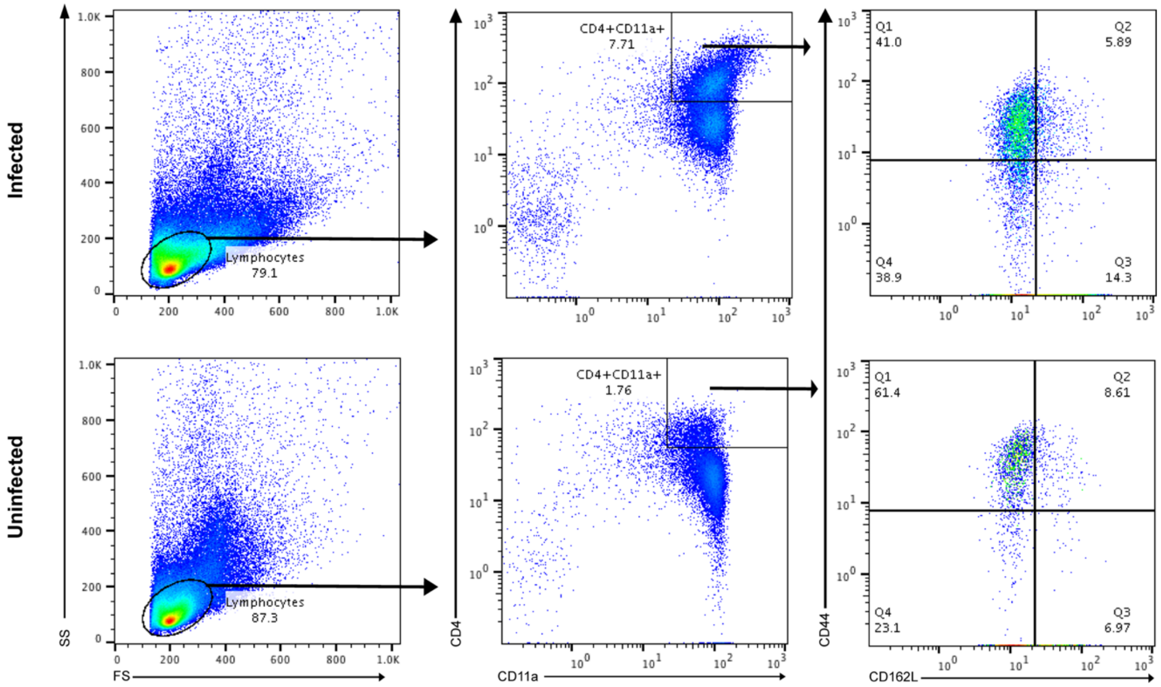

Additional File Figure 1: Flow cytometer gating strategy for Effector CD4 T cells (Teff) activation. Spleen cells were obtained from *P. chabaudi* infected (top panels) or uninfected Moringa-treated mice (bottom panels) and stained for CD4, CD11a, CD44, CD62L. Lymphocytes were identified by side and forward scatter (left panel). Recently activated CD4 T cells were identified as $CD4^{+}CD11a^{+}$ (middle panel) and effector T cells were identified as $CD44^{hi}CD62L^{lo}$ (right panel).

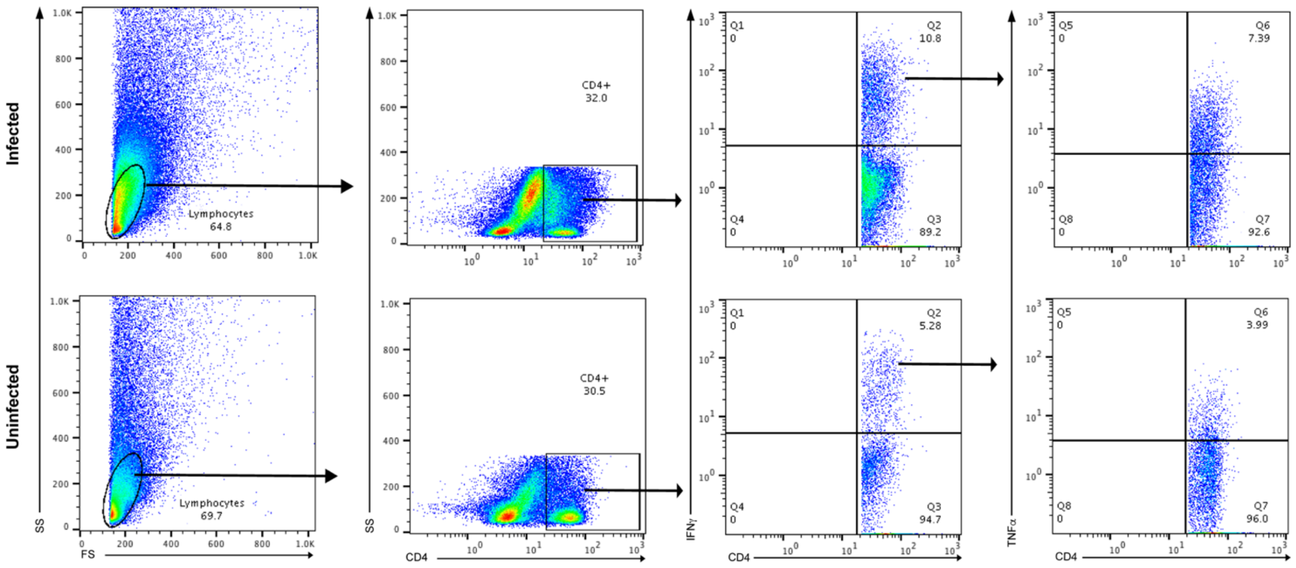

Additional File Figure 2: Flow cytometer gating strategy for IFNγ secreting CD4 T cells. Spleen cells were obtained from *P. chabaudi* infected (top panels) or Moringa treated uninfected (bottom panels) mice. Cells were stimulated for intracellular stained as explained in the *materials and methods* section. Lymphocytes were identified by side and forward scatter (left panels) and IFNγ and TNFα secreting cells CD4 T cells were identified by $CD4^{+}IFN\gamma^{+}$ or $CD4^{+}TNFa^{+}$ (two panels to the right).

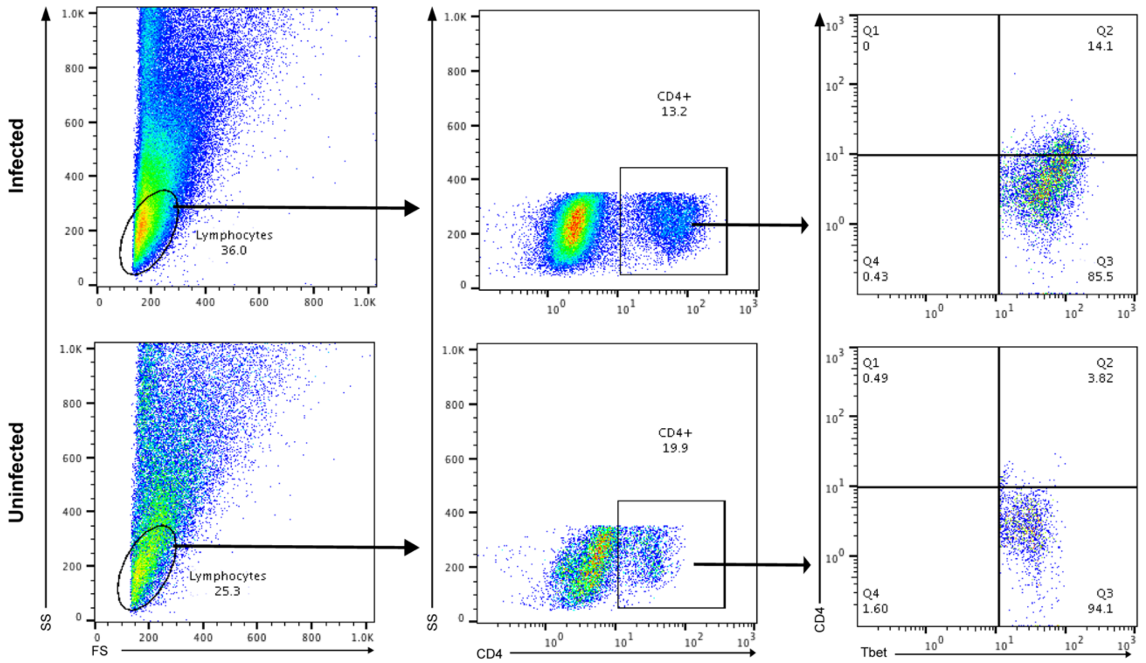

Additional File Figure 3: Flow cytometer gating strategy for Tbet expression byCD4 T cells. Spleen cells were obtained from adult *P. chabaudi* infected (top panels) or uninfected Moringa-treated mice (bottom panels). Cells were surface stained for CD4, fixed in 2% paraformaldehyde then permeabilized using permeabilization/FOXP3 buffer and intracellularly stained for Tbet. Lymphocytes were identified by side and forward scatter (left panels), followed by Tbet expression on CD4 cells identified as $CD4^{+}Tbet^{+}$ (middle and right panels).
